# Consensus statements for the transdisciplinary care of patients with epidermolysis bullosa in South Africa: Part 1

**DOI:** 10.4102/hsag.v30i0.2963

**Published:** 2025-08-06

**Authors:** Antoinette V. Chateau, Carol Hlela, Ncoza Dlova, Thuraya Isaacs, Thirona Naicker, Tracey Nupen, Julia Ambler, Frans Maruma, Lushen Pillay, Avumile Mankahla, Fatima Moosa, Jabulile Makhubele, Rannakoe Lehloenya, Willem I. Visser, Caridad Velazquez, Sinead Cameron-Mackintosh, Gail Todd, David Blackbeard, Colleen Aldous

**Affiliations:** 1 Department of Dermatology, Faculty of Health Sciences, Greys Hospital, Pietermaritzburg, South Africa; 2 Department of Dermatology, Faculty of Health Sciences, University of KwaZulu-Natal, Durban, South Africa; 3 Department of Dermatology, Faculty of Health Sciences, Red Cross Children’s Hospital, Cape Town, South Africa; 4 Department of Dermatology, Faculty of Health Sciences, University of Cape Town, Cape Town, South Africa; 5 Department of Pediatrics, Clinical Genetics, Faculty of Health Sciences, University of KwaZulu-Natal, Durban, South Africa; 6 Department of Paediatric and Child Health, Faculty of Health Sciences, University of Cape Town, Cape Town, South Africa; 7 Paedspal Non-Profit Organisation, Cape Town, South Africa; 8 Umduduzi Hospice Care for Children, Durban, South Africa; 9 Department of Family Medicine, Faculty of Health Science, University of KwaZulu-Natal, Durban, South Africa; 10 Department of Pediatrics, Faculty of Health Sciences, University of KwaZulu-Natal, Durban, South Africa; 11 Department of Dermatology, Faculty of Health Sciences, University of the Free State, Bloemfontein, South Africa; 12 Department of Dermatology, Faculty of Health Sciences, University of the Witwatersrand, Johannesburg, South Africa; 13 Department of Internal Medicine and Pharmacology, Nelson Mandela Academic Hospital, Umtata, South Africa; 14 Department of Internal Medicine and Pharmacology, Walter Sisulu University, Umtata, South Africa; 15 Private practice, The Derm Factor, Bloemfontein, South Africa; 16 Department of Dermatology, Faculty of Health Sciences, University of Pretoria, Pretoria, South Africa; 17 Division of Dermatology, Faculty of Health Sciences, University of Cape Town, Cape Town, South Africa; 18 Division of Dermatology, Department of Medicine, Faculty of Medicine and Health Sciences, Stellenbosch University, Cape Town, South Africa; 19 Department of Anaesthesiology and Pain Management, Faculty of Health Sciences, Greys Hospital, Pietermaritzburg, South Africa; 20 Simply Genetics, Hilton, KwaZulu-Natal, South Africa; 21 Department of Medicine, Faculty of Health Sciences, University of Cape Town, Cape Town, South Africa; 22 Department of Clinical Psychology, Faculty of Health Sciences, Greys Hospital, Pietermaritzburg, South Africa; 23 Department of Psychiatry, Faculty of Health Sciences, University of KwaZulu-Natal, Durban, South Africa; 24 School of Clinical Medicine, University of KwaZulu-Natal, Durban, South Africa

**Keywords:** epidermolysis bullosa, diagnosis, clinical care, complications, transdisciplinary care, palliative care, pain, pruritus, consensus recommendations, prevention, genetic counselling

## Abstract

**Background:**

Epidermolysis bullosa (EB) is a rare, painful and blistering genodermatosis with no cure. Treatment aims to prevent new lesions and manage complications. Previously, there were no management guidelines or consensus recommendations for patients with EB in Africa.

**Aim:**

The aim of this study is to produce a comprehensive, transdisciplinary and practical care guide that is contextually appropriate to the cultural setting and resource limitations in South Africa.

**Setting:**

Multicentre, multiprovincial study involving healthcare practitioners from five South African provinces – KwaZulu-Natal, Gauteng (Johannesburg, Pretoria), Western Cape (Cape Town, Stellenbosch), Free State (Bloemfontein) and the Eastern Cape (Umtata).

**Methods:**

Consensus recommendations for the care of patients with EB were developed by a transdisciplinary team of specialists in consultation with EB patients. The modified Delphi technique was used to reach a robust consensus with a threshold of 80% for each action point to ensure the validity and reliability of the recommendations.

**Results:**

In all, 16 consensus statements were developed, and the main themes included the clinical clues to the diagnosis, complications as per the subtype of EB, diagnostics in a resource-limited environment, management of EB, pruritus and pain, palliative care and genetic counselling.

**Conclusion:**

A transdisciplinary approach is essential for the holistic care of patients and their families with EB in the context of their resource limitations and cultural diversity providing much-needed guidance for clinicians in South Africa and similar settings.

**Contribution:**

This is the first consensus recommendation of care for patients with EB in Africa.

## Introduction

### Background

Epidermolysis bullosa (EB) is a rare, incurable and painful inherited skin condition resulting in skin fragility with over 30 clinical variants and 21 implicated genes. Various proteins maintain the integrity of the skin (Has et al. 2020a). There are four major types of EB, presenting as localised or generalised disease depending on the location of the mutations of these proteins and hence the level of cleavage: EB simplex (intraepidermal), junctional EB (lamina lucida), dystrophic EB (sub lamina densa) and Kindler syndrome (mixed cleavage planes) (Has et al. 2020a). Complications include sepsis, mucocutaneous fragility, scarring, dystrophy of the nails and other ectodermal structures, gastrointestinal, genitourinary, ocular cardiac, renal and orthopaedic, skin cancers, psychological affectation, and can result in death (Fine & Mellerio 2009a, 2009b).

Epidemiology varies per country, with the incidence being 67.6 per million live births in England as opposed to 45.0 per million live births in Germany and 19.57 per million live births in the United States (US), with a prevalence of 34.8 per million in England, 54.8 per million in Germany and 11.07 per million in the US (Fine 2016; Has et al. 2023; Petrof et al. 2022). In South Africa (SA), there is no EB registry and no known epidemiological data. Currently, there are no consensus statements or guidelines for the care of EB patients in Africa.

Being a rare genodermatosis, not all healthcare practitioners (HCPs) are familiar with EB. This results in a delay in diagnosis and management, which may result in mortality and morbidity. Clinically diagnosing EB can be challenging, as accurate subtype determination is essential for managing associated complications, guiding treatment and providing genetic counselling. As EB is an inherited condition, knowing the subtype will allow for accurate genetic counselling as to the possibility of a couple having another baby with the same condition. The diagnosis and management of EB is expensive and unaffordable for many patients with EB in SA, and the condition can profoundly affect the quality of life of the patient and their families. Diagnosis is based on clinical features as well as investigations which include electron microscopy, immunofluorescent mapping and genetic testing such as whole exome sequencing. The reagents are extremely expensive, and no laboratories in the private and public healthcare sector are set up to diagnose EB with all modalities, including electron microscopy, a full panel of immunofluorescence mapping and whole exome sequencing.

There is no cure for EB and treatment is based on preventing new blister formation, wound care, relieving symptoms and preventing and treating complications (Pope et al. 2012).

There is a paucity of clinical trials to date and care is guided by expert opinion, consensus recommendations and guidelines from Dystrophic Epidermolysis Bullosa Research Association (DEBRA) International (DEBRA International 2017; El Hachem et al. 2014a; Has et al. 2020a; Pope et al. 2012). International guidelines make no mention of the traditional and cultural beliefs that are integral to providing holistic care for patients and few guidelines consider the financial constraints and hence inaccessibility of diagnostics and essential dressings.

International literature has documented and local experience in SA has noted that not all HCPs are *au fait* with this complex rare disease (Chateau et al. 2024a, 2024b). The modified Delphi method was used to determine consensus among South African specialists knowledgeable in EB in order to develop consensus statements for the diagnosis and comprehensive treatment of EB as a step towards improving clinical practice for patients with EB.

The aim of this study was to develop two consensus statements documents, Part 1 and Part 2, which will incorporate all facets of care for patients with EB. Part 1 focuses on clinical and laboratory diagnostics, wound care, pain, pruritus management, palliative care and genetic counselling. Part 2 focuses on the biopsychosocial-cultural aspect with the patient at the centre of care. Many of our African patients seek the help of traditional healthcare practitioners before seeking allopathic healthcare. It is important to be cognisant of a patient’s cultural beliefs and practices. Understanding that parents and patients may be advised to perform certain practices that may exacerbate the condition, thus being aware of these practices may prevent complications. This emphasises the need to be cognisant of patients’ cultural beliefs and practices in the comprehensive care of patients (Chateau et al. 2023). Part 2 included care by paediatricians, an ophthalmologist, a gynaecologist, a dentist and allied healthcare professionals. These consensus statements aim to empower HCPs to accurately diagnose, manage and refer patients with EB and to improve outcomes and the quality of life of patient and their families.

Given the complexity and rarity of EB, as well as the challenges faced by patients and families in resource-limited settings like SA, there is an urgent need for a structured approach to developing consensus statements for our local setting.

The aim is to produce comprehensive, multidisciplinary consensus statements of care that are cognisant of the cultural setting and resource limitations in SA.

## Research methods and design

A core task team consisting of dermatologists and paediatricians, as they are usually the primary care providers in the team, met to define the strategy, structure and important topics that would be incorporated into the consensus statements for the comprehensive care of patients with EB. It was decided that there would be two documents for this comprehensive review. As many as 19 specialists were invited to partake in part 1 of the consensus statements and 20 specialists were invited to take part in part 2, and all accepted and consented to participate in this research. The group met on the 13th December 2022 via Zoom^®^ to discuss the vision of the task team. The transdisciplinary research team including dermatologists invited from each province in SA via the Dermatology Society of South Africa; and other specialists in KwaZulu-Natal including paediatricians, emergency care specialists, palliative care and pain specialists, clinical psychologists, an orthopaedic surgeon, ophthalmologist, dietician, occupational therapist, physiotherapist gynaecologist, genetics counsellor and a social worker, a dentist and a podiatrist were invited to participate in the study. Each team drafted specific questions on their assigned topic within their speciality, which were circulated via a Google Form, to achieve initial consensus using a 4-point Likert scale, that is, strongly disagree, disagree, agree or strongly agree. These topics were then reviewed by patients with EB through DEBRA SA to ensure relevance and practicality. The patients were included in subsequent rounds of discussion.

A transdisciplinary methodology included a review of existing literature, consultations with experienced EB HCPs and engagement with families to understand their needs and preferences. Literature searches including EB-CLINET guidelines (https://www.eb-clinet.org/clinical-guidelines/), DEBRA international resources (https://www.debra-international.org/) and databases such as PubMed, EBSCOhost, Google Scholar, Clinical Key and Wiley. This multifaceted approach ensures that the consensus statements are clinically relevant, culturally sensitive and financially feasible.

A modified Delphi technique was employed to reach a robust consensus, involving three rounds of discussion. The Accurate Consensus Reporting Document (ACCORD) using a modified Delphi technique by Gattrell et al. (2004) was followed. A consensus threshold of 80% was set for each action point to ensure the validity and reliability of the recommendations. All 16 consensus statements were accepted and were included in Part 1 of the document.

This care guide was developed for all healthcare professionals who manage and support patients and families affected by EB in SA.

### Ethical considerations

An application for full ethical approval was made to the Biomedical Research Ethics Committee of University of KwaZulu-Natal and consent was received on 7 May 2022 (reference no: BREC/00003768/2022).

## Results

The results of the consensus statements are summarised in Table 1.

**TABLE 1:** Overview of the consensus statements.

Consensus recommendations	Total (%)	References
**A. History and examination**		
*Consensus statement 1* Thorough history and examination in EB diagnosis	100	Martinez-Moreno et al. (2020)
*Consensus statement 2* Identifying clinical clues to delineate EB subtypes	94	Krämer et al. (2020), Yenamandra et al. (2017b), Has et al. (2020b, 2021), Pope et al. (2012), Has and Fischer (2019), Fine et al. (2014)
**B. Complications**		
*Consensus statement 3*		
Monitoring and detecting cutaneous and extracutaneous complications by EB subtype	100	Fine et al. (2008), Fine and Mellerio (2009a, 2009b), El Hachem et al. (2014), Boeira et al. (2013)
**C. Diagnosis**		
*For the laboratory diagnosis of EB we recommend*		
In a resource-limited environment, immunohistochemistry or IFM with a limited panel is recommended as an alternative	94	Yenamandra et al. (2017a)
In a resource-rich environment, we strongly recommend IFM, EM and genetic testing	100	Has et al. (2020b), Pohla-Gubo, Cepeda-Valdes and Hintner (2010), Mariath et al. (2020)
*Consensus statement 5*		
We recommend identifying the correct biopsy site and technique and preservation medium based on the required investigation.	100	Has et al. (2020b)
**D. Management**		
*Consensus statement 6*		
We recommend preparing the hospital/clinic environment to minimise trauma in EB.	94	Abreu Molnar et al. (2024)
*Consensus statement 7*		
We recommend measures to prevent wound formation.	100	El Hachem et al. (2014), Saad et al. (2024), Abreu Molnar et al. (2024)
*Consensus statement 8*		
We recommend regularly assessing wounds for complications such as infection and malignancy.	100	Pope et al. (2012), Sibbald, Woo and Ayello (2006), Mellerio et al. (2016), El Hachem et al. (2014)
*Consensus statement 9*		
We recommend creating an ideal environment for wound healing.	100	Fonder et al. (2008), Liy-Wong et al. (2023), Pope et al. (2012)
*Consensus statement 10*		
We recommend that all blisters are lanced and not deroofed.	100	Denyer, Pillay and Clapham (2017)
*Consensus statement 11*		
*Bathing recommendations for patients with epidermolysis bullosa*		
Bath additives: Bleach (sodium hypochlorite) salt, acetic acid and 0.1% chlorhexidine scrubs	88	Petersen et al. (2015), Shayegan et al. (2020), El Hachem et al. (2014), Pope et al. (2012), Madhusudhan (2016)
The frequency of bathing depends on the dressings used; bathing on average every 2–3 days is advised.	88	El Hachem et al. (2014), Pope et al. (2012), Denyer and Stevens (2010)
Method of washing the skin: bathing is recommended over showering	88	Arbuckle (2010), DEBRA International
*Consensus statement 12: Wound care recommendations for EB*		
Specific dressings to use in a resource-limited and resource-rich environment	100	El Hachem et al. (2014), Pope et al. (2012), Has et al. (2021), Denyer et al. (2017), DEBRA International
Frequency of dressing changes Dressings used in a resource-limited environment should be changed daily. More advanced dressings can be changed every 3–4 days, up to 7 days.	94	Shayegan et al. (2020), Pope et al. (2012)
Topicals that can be used Antibiotics for infected skin Topical steroids for over granulated wounds Emollients for xerotic skin Sunscreen for patients with Kindler syndrome	94	Has et al. (2021), El Hachem et al. (2014)
*Consensus statement 13: Understanding pain mechanisms to guide analgesia choice*		
Assess the mechanisms of pain: nociceptive or neuropathic	94	Pope et al. (2012), Has et al. (2021), Goldschneider et al. (2014), El Hachem et al. (2014)
Therapeutic options for pain: acute, chronic, psychological and procedural pain		
*Consensus statement 14: We recommend a biopsychological approach to managing pruritus*		
Lifestyle modification Topical therapy Systemic therapy Behavioural or psychological modification	100	Pope et al. (2012), Papanikolaou et al. (2021), El Hachem et al. (2014)
*Consensus statement 15: Palliative care is an essential aspect of EB management Multidisciplinary approach to care*		
Encourage effective communication Ethical consideration and shared decision-making Anticipating and managing complicated grief	–	Goldschneider et al. (2014), Popenhagen et al. (2023), Larcher et al. (2015)
*Consensus statement 16: Genetic counselling is essential to holistic care for patients and families*		
Educate and empower patients and parents about the condition and the genetic results Adjust realistic expectations Provide emotional support and palliative care referrals Address recurrence risk for subsequent pregnancies	–	Chong et al. (2021)

Note: Please see full reference list of this article: Chateau, A.V., Hlela, C., Dlova, N., Isaacs, T., Naicker, T., Nupen, T. et al., 2025, ‘Consensus statements for the transdisciplinary care of patients with epidermolysis bullosa in South Africa: Part 1’, *Health SA Gesondheid* 30(0), a2963. https://doi.org/10.4102/hsag.v30i0.2963 for more information.

IFM, immunofluorescent mapping; EB, epidermolysis bullosa; EM, electron microscopy.

### History and examination

#### Consensus statement 1: Thorough history and examination in epidermolysis bullosa diagnosis

History

- Assess maternal history of EB or blistering disease, polyhydramnios or infections.
- Identify any family history of blistering or skin fragility.
- Note the presence of vesicles, bullae or skin fragility in the patient.

Examination

- Rule out other causes of blisters such as infections, autoimmune blistering disease, epidermolysis ichthyosis, bullous insect bite or inflammatory conditions such as mastocytosis.
- Inspect skin, hair, nails and mucosa – to determine the extent and location of lesions.
- Look for congenital absence of skin.
- Conduct a systemic examination, focusing on respiratory, cardiovascular, abdominal, neurological and genitourinary systems.

Patients with EB may present at birth with blisters, vesicles or congenital absence of skin, often accompanied by mucosal erosions. Congenital skin absence can occur in all EB types; and when linked with pyloric atresia in junctional EB indicates a poor prognosis (Martinez-Moreno et al. 2020). Systemic complications include duodenal atresia, nephrotic syndrome, cardiomyopathy, respiratory distress, muscular dystrophy, and genitourinary anomalies, may also be present.

#### Consensus statement 2: Identifying clinical clues to delineate epidermolysis bullosa subtypes

Delineating the specific subtype of EB in the neonatal period is often challenging; however, clinical clues can help differentiate the four main EB subtypes, as outlined in Table 2 (Has et al. 2021; Krämer et al. 2020; Pope et al. 2012; Yenamandra et al. 2017b).

**TABLE 2:** Mucocutaneous and ectodermal clues to the clinical diagnosis of the four subtypes of epidermolysis bullosa.

Clinical features	EB Simplex (EBS)	Junctional EB (JEB)	Dystrophic EB (DEB)	Kindler
Specific characteristics	Herpetiform lesions in EBS generalised severe Natal teeth Acral lesions in the localised types	Extensive granulation tissue of skin, mucosa and nail bed	Absence of lingual papillae in recessive dystrophic EB (RDEB) Pseudosyndactyly with mitten deformity in RDEB	Poikiloderma and photosensitivity Digit webbing/partial pseudosyndactyly
Mucocutaneous	Extensive skin and mucous membrane fragility Improves with age No scarring Heals with dyspigmentation -No chronic wounds	Extensive skin and mucous membrane involvement Hoarse cry and possible respiratory distress Chronic wounds Failure to thrive	Dominant dystrophic – localised Recessive dystrophic – generalised Extensive skin and mucous membrane involvement Heal with scarring and milia Chronic wounds	Erosions and vesicles in early infancy improve with age Atrophic scaring No chronic wounds
Hair	No alopecia	Scarring alopecia	Scarring alopecia	No alopecia
Nails	Transiently affected then regrows	Dystrophic nails	Dystrophic nails	Dystrophic nails

*Source*: Has, C., El Hachem, M., Bučková, H., Fischer, P., Friedová, M., Greco, C. et al., 2021, ‘Practical management of epidermolysis bullosa: Consensus clinical position statement from the European reference network for rare skin diseases’, *Journal of the European Academy of Dermatology and Venereology* 35(12), 2349–2360. https://doi.org/10.1111/jdv.17629; Krämer, S., Fuentes, I., Yubero, M.J., Encina, C., Farfán, J., Araya, I. et al., 2020, ‘Absence of tongue papillae as a clinical criterion for the diagnosis of generalized recessive dystrophic epidermolysis bullosa types’, *Journal of the American Academy of Dermatology* 83(6), 1815–1816. https://doi.org/10.1016/j.jaad.2020.03.117; Pope, E., Lara-Corrales, I., Mellerio, J., Martinez, A., Schultz, G., Burrell, R. et al., 2012, ‘A consensus approach to wound care in epidermolysis bullosa’, *Journal of the American Academy of Dermatology* 67(5), 904–917. https://doi.org/10.1016/j.jaad.2012.01.016; Yenamandra, V.K., Moss, C., Sreenivas, V., Khan, M., Sivasubbu, S., Sharma, V.K. et al., 2017b, ‘Development of a clinical diagnostic matrix for characterizing inherited epidermolysis bullosa’, *British Journal of Dermatology* 176(6), 1624–1632. https://doi.org/10.1111/bjd.15221

Yenamandra et al. developed a clinical diagnostic matrix that is particularly useful in resource-limited settings. The approach supports EB diagnosis when advanced techniques such as immunofluorescent mapping (IFM), electron microscopy (EM) or whole exome sequencing (WES) is unavailable (Yenamandra et al. 2017b).

There are currently over 30 variants of EB currently. Online Appendix 1 – Table 1-A1 to Table 8-A1 provide the detailed information on the clinical features, inheritance patterns, target proteins and implicated genes for the major EB subtypes (Fine et al. 2014; Has & Fischer 2019; Has et al. 2020b).

### Complications

#### Consensus statement 3: Monitoring and detecting cutaneous and extracutaneous complications by epidermolysis bullosa subtype

Epidermolysis bullosa presents with diverse complications, ranging from skin-limited issues to systemic, potentially life-threatening conditions. Identifying the specific EB subtype is crucial for preventing complications, many of which may be preventable or treatable if detected early, thereby reducing long-term consequences.

Mortality rates differ significantly among EB subtypes, underscoring the importance of accurate diagnosis. A study done by Fine et al. found that early mortality in junctional EB (JEB) often results from sepsis, failure to thrive and respiratory failure. In epidermolysis bullosa simplex (EBS) generalised-severe subtype, sepsis is the primary cause of death. In recessive dystrophic EB (RDEB), squamous cell carcinomas (SCCs) are the leading cause of mortality later in life (Fine et al. 2008). Table 3 provides a comprehensive summary of complications associated with various EB subtypes (Boeira et al. 2013; Fine & Mellerio 2009a, 2009b, El Hachem et al. 2014).

**TABLE 3:** Complications according to subtypes of epidermolysis bullosa.

Organ systems	Clinical features	EB subtypes
General	Pain	JEB, DEB, EBS, KEB
	Infections	JEB, DEB, EBS
	Poor wound healing	JEB, DEB, EBS
**Integument (skin, hair and nails)**		
Nails	Onychodystrophy	EBS, JEB, DEB
	Atrophic nails	JEB, DEB
	Parrot beak nail deformity	KEB
	Periungual or subungual blistering	EBS, JEB, DEB
Hair	Cicatricial alopecia	JEB, DEB
Skin	Poikiloderma	Kindler
	Scarring - atrophy and pigmentation	EBS, JEB, DEB
	Hypertrophic scarring	DEB, RDEB
	Excessive granulation tissue	LOC, JEB
	Congenital absence of skin (aplasia cutis)	JEB, DEB, EBS, KEB
	EB nevi	EBS, JEB, DEB
	Milia	EBS-DM, JEB, DEB, KS
	Palmoplantar keratoderma	EBS mainly, JEB, DEB
	Albopapuloid lesions	DEB
Ocular	Injected red eyes	JEB, RDEB EBS,
	Subconjunctival haemorrhage	JEB
	Corneal blistering, erosions and scarring	JEB, RDEB
	Ectropion and corneal drying	JEB
	Symblepharon	JEB, RDEB
	Granulation tissue and lacrimal duct obstruction	RDEB
	Conjunctival granulation tissue	LOC
Oral and dental	Intraoral blisters and erosions	EBS, JEB, DEB, KEB
	Microstomia and ankyloglossia	RDEB
	Inability to suckle	RDEB-HS, JEB
	Absent lingual papillae, milia	RDEB
	Severe oral blistering	KEB
	Enamel pitting/hypoplasia, increased dental caries	JEB
	Premature loss of dentition	JEB, RDEB-HS
	Microstomia and ankyloglossia	RDEB, JEB
	Periodontitis	Kindler
**Ear, nose, throat and upper airways**		
Ear	Blisters, erosions of external auditory canal	JEB, RDEB
	Otitis media and externa	JEB, RDEB
	Sensorineural deafness	RDEB
Nose	Erosions and granulation tissue of nostrils	JEB, LOC
Oropharynx	Blisters and erosions of oropharynx	JEB, DEB
Tracheolaryngeal	Stenosis	JEB, EBS-DM, EBS muscular dystrophy, RDEB
	Excessive granulation tissue	Laryngo-onycho-cutaneous syndrome
Gastrointestinal tract	Oesophageal erosions and blistering	EBS, JEB, RDEB,
	Gastroesophageal reflux disease	EBS, JEB, RDEB,
	Web formation, stenosis, strictures, hiatus hernia	RDEB
	Rectal tears, prolapse, perianal fistulae	JEB, DEB
	Pyloric atresia	EBS, JEB
	Growth retardation- increased expenditure, decrease intake, malabsorption May need gastrostomy	JEB, RDEB
	Constipation	EBS, JEB, RDEB
	Inflammatory bowel disease	EBS, JEB
Genitourinary	Renal insufficiency and failure	RDEB
	Renal amyloidosis	RDEB
	Uroendothelium blister formation, scarring and resultant bladder retention, hydronephrosis	JEB, JEB-PA, RDEB
Cardiopulmonary	Cardiomyopathy	RDEB-HS, JEB, ESB-MD
	Viral myocarditis	RDEB
Musculoskeletal	Pseudosyndactyly (mitten deformity)	RDEB mainly, JEB, DDEB, EBS-DM
	Contractures of hands and feet	RDEB, JEB, DDEB
	Osteopenia, osteoporosis	RDEB, JEB, EBS-DM
	Muscle dystrophy	EBS with muscle dystrophy
	Acroosteolysis	RDEB
	Thoracic and thoracolumbar scoliosis	RDEB
	Webbing of digits or partial pseudosyndactyly	Kindler
Nutritional	Micronutrient (selenium deficiency)	JEB, RDEB
	Vitamin D deficiency	JEB, RDEB
	Failure to thrive	EBS, JEB, DEB
	Hypoalbuminemia	JEB, RDEB
	Malabsorption	JEB, RDEB
Endocrine	Delayed puberty	RDEB
Haematologic	Anaemia	EBS, JEB, DEB
Psychological	Depression and anxiety Strained family dynamic	EBS, JEB, DEB, KEB
Malignancy	SCC - skin, tongue, oesophagus	RDEB-HS, JEB, Kindler
	BCC	EBS-DM
	Melanoma	RDEB-HS

*Source*: Boeira, V.L., Souza, E.S., Rocha Bde, O., Oliveira, P.D., Oliveira Mde, F., Rêgo, V.R. et al., 2013, ‘Inherited epidermolysis bullosa: Clinical and therapeutic aspects’, *Anais Brasileiros de Dermatologia* 88(2), 185–198. https://doi.org/10.1590/S0365-05962013000200001; El Hachem, M., Zambruno, G., Bourdon-Lanoy, E., Ciasulli, A., Buisson, C., Hadj-Rabia, S. et al., 2014, ‘Multicentre consensus recommendations for skin care in inherited epidermolysis bullosa’, *Orphanet Journal of Rare Diseases* 9, 76–76. https://doi.org/10.1186/1750-1172-9-76; Fine, J.-D. & Mellerio, J., 2009a, ‘Extracutaneous manifestations and complications of inherited epidermolysis bullosa Part I. Epithelial associated tissues’, *Journal of the American Academy of Dermatology* 61(3), 367–384. https://doi.org/10.1016/j.jaad.2009.03.052; Fine, J.-D. & Mellerio, J., 2009b, ‘Extracutaneous manifestations and complications of inherited epidermolysis bullosa. Part II. Other organs’, *Journal of the American Academy of Dermatology* 61(3), 387–402. https://doi.org/10.1016/j.jaad.2009.03.053

EBS, epidermolysis bullosa simplex; JEB, junctional epidermolysis bullosa; DEB, dystrophic epidermolysis bullosa; RDEB, recessive dystrophic epidermolysis bullosa; DDEB, dominant dystrophic epidermolysis bullosa; EBS-DM, epidermolysis bullosa simplex Dowling Meara subtype; KEB, Kindler; LOC, laryngo-onycho-cutaneous syndrome; EBS-MD, epidermolysis bullosa-Muscular dystrophy; EB, epidermolysis bullosa; BCC, basal cell carcinoma; SCC, squamous cell carcinoma; KS, Kindler syndrome; RDEB-HS, RDEB Hallopeau–Siemens subtype; JEB-PA, junctional epidermolysis bullosa with pyloric atresia.

### Diagnosis

#### Consensus statement 4: For the laboratory diagnosis of epidermolysis bullosa, we recommend

**A systematic approach to diagnosis in resource-limited environments:** Accurate diagnosis and subclassification of EB are critical for early prognostication, monitoring for complications, guiding management decisions and informing genetic counselling. The clinical heterogeneity of EB, especially neonates and mild forms, necessitates laboratory diagnostics as clinical features alone are often insufficient (Has et al. 2020b).

In SA, absence of dedicated diagnostic centres limits expertise. Diagnosis often relies on clinical evaluation supplemented by haematoxylin and eosin (H&E) tissue staining and occasionally, transmission electron microscopy. However, the DEBRA guidelines recommend sequential use of IFM, EM and genetic testing for accurate diagnosis and subclassification (Has et al. 2020b; Pohla-Gubo et al. 2010). However, these tools are not widely available locally.

#### Resource-limited settings

**Haematoxylin and eosin:** While H&E is not helpful for diagnosing EB subtypes, it aids in excluding other vesicular or bullous disorders.

**Immunohistochemistry:** Immunohistochemistry (IHC), available in most pathology laboratories, in an affordable alternative to IFM. Unlike IFM, IHC uses peroxidase-tagged antibodies eliminating the need for fluorescent microscope.

**Frozen sections:** Using four key antibodies (cytokeratin-14, laminin-332, collagen IV and collagen VII), IHC on frozen sections can accurately identify major EB subtypes, achieving 80% concordance with genetic testing (Yenamandra et al. 2017a). It also provides a better visualisation of epidermal architecture compared to the IFM. Furthermore, IHC had the advantage of showing changes in the epidermal architecture that were not that appreciable on IFM. Hence, IHC using frozen sections can be useful in resource-limited settings where facilities for other diagnostic tests such as IFM, EM and genetic testing are unavailable.

**Formalin-fixed paraffin-embedded specimens:** Regrettably, IHC on formalin-fixed paraffin-embedded specimens is less reliable because of inconsistent staining and antigens loss during processing, thus making it unsuitable for EB diagnosis (Has et al. 2020b).

#### Resource-rich environments

**Immunofluorescence mapping:** Antibody staining for keratin 14 and collagen IV is highly sensitive and specific to diagnose EB (Has et al. 2020b).

Furthermore, IFM on frozen sections is the recommended first step for diagnosing neonates with suspected EB because of its speed and accuracy (Has et al. 2020b). Key antibodies include collagen IV, VII, XVII, keratin 14 and laminin β3. A full panel is detailed in the guidelines by Has et al. (2020b).

**Transmission electron microscopy:** Transmission electron microscopy the original diagnostic tool for EB, remains useful for detecting milder forms (e.g., EB simplex-generalised severe, autosomal recessive EB simplex, where IFM may appear normal (Has et al. 2020b).

However, EM is expensive, time-intensive, and operator-dependent, thus limiting its accuracy (Pohla-Gubo et al. 2010).

#### Genetic testing

Genetic testing is essential for all patients with suspected or confirmed EB (Has et al. 2020b; Mariath et al. 2020).

Methods include the following:

- Sanger sequencing for known genes.
- Gene panels covering the 21 identified EB-related genes.
- Whole exome sequencing for novel mutations or unknown genes.

#### Consensus statement 5: We recommend identifying the correct biopsy site and technique and preservation medium based on the required investigation

**Taking the biopsy and storing the sample (Has et al. 2020b) -** Select a non-sun-exposed site:

- Induce a blister using the rubber-end of a pencil, an earbud (Q-tip) or a pus swab (common in SA).
- Avoid topical anaesthetics, as they may cause artefacts.
- Perform a 4mm – 6mm punch or wedge biopsy, avoiding shear forces that could distort the specimen’s architecture.
- Store skin samples as follows: Michel’s transport medium for IFM, glutaraldehyde for EM and formalin for H&E staining. Samples remain at room temperature but should be sent to the laboratory promptly.

#### Genetic testing

- Collect two 5 ml blood samples in EDTA tubes.
- Transport on ice to a laboratory promptly and store at -80 °C.

### Management

#### Management of patients with epidermolysis bullosa in a resource-limited setting

There is no cure for EB; however, management focuses on preventing new blisters and treating wounds. Wound care is particularly challenging in a resource-constrained environment such as SA, where dressing changes are costly and time-consuming, significantly impacting quality of life for patients’ and their families.

#### Consensus statement 6: We recommend preparing the hospital/clinic environment to minimise trauma in epidermolysis bullosa

For a mother about to deliver a neonate with suspected or confirmed EB (Abreu Molnar et al. 2024):

- Notify the multidisciplinary team in advance.
- Arrange and prepare the delivery room and nursery to minimise trauma.
- Implement measures to prevent blister formation (refer to consensus statement 7).
- Ensure the neonate is promptly transferred to an EB specialist unit.

Detailed preparation and management for pregnant patients with EB during delivery will be discussed in Part 2 of these consensus recommendations.

#### Consensus statement 7: We recommend measures to prevent wound formation

Preventing blister formation is crucial in managing EB. Strategies to minimise wound formation are detailed in Table 4 (Abreu Molnar et al. 2024; El Hachem et al. 2014; Saad et al. 2024).

**TABLE 4:** Preventing wound formation.

Possible triggers of wound formation	Management strategies to prevent wound formation
Environment	Do not routinely place the baby in an incubator, as heat induces blisters.
Name tag	Place name tags on the clothing or cot instead of the wrist.
Umbilical cord	Ligate instead of placing a clamp on the umbilical cord.
Handling	Encourage kangaroo care, encourage contact for bonding and soothing. Place your hand below the baby’s head and buttocks when lifting the baby.
Monitoring	No unnecessary probes. Apply padding below the blood pressure cuff. Infrared thermometers or lubricate the thermometer that is placed in the axilla (in a resource-limited setting). Remove the adhesive tape from the electrodes and fix them with silicone tape. Clip sensors for pulse oximetry. Avoid suctioning; if necessary, use the shortest and most flexible tube with minimal suction pressure. When removing electrodes, use 50/50 white soft paraffin/liquid paraffin (50/50 WSP in LP).
Procedures	Nasogastric tubes, drips and catheters secured with silicone tape or micropore. Umbilical venous catheter access in sick neonates. Padding below the tourniquet when drawing blood if indicated in older patients.
Traditional practices	Respectfully caution parents regarding traditional scarification, fastening traditional symbolisms such as tying bones and coins on a string around the baby’s waist, applying traditional herbs onto the skin, and inserting herbal enemas.
Bathing	See bathing recommendations below. The frequency of bathing is individually determined. Infected wounds: add chlorhexidine (0.1%) or acetic acid to the bath water; see the table below. Clean the diaper area gently with cotton wool and 50/50 white soft paraffin in liquid paraffin.
Clothing	Turn the clothing inside out, as labels can induce blisters. Napkins should be of the correct size and lined with soft material such as silicone contact layer or foam, paraffin-impregnated gauze, hydrogel-impregnated gauze such as Intrasite conformable. Shoes must be soft and comfortable shoes with no inner seam.
Feeding	Breast: apply petroleum jelly on the baby’s lips and mother’s breast to prevent friction. Bottle: heat the teat and increase the size of the hole of the teat to prevent trauma from sucking. Nasogastric feeds: insert a soft polyurethane tube and secure it with silicone tape.

*Source*: Abreu Molnar, B., Levin, L., Yun, D., Morel, K., Wiss, K., Wieser, J. et al., 2024, ‘Inpatient management of epidermolysis bullosa: Consensus-based hands-on instructions for neonates and postneonates’, *Journal of the American Academy of Dermatology* 91(2), 290–299. https://doi.org/10.1016/j.jaad.2024.04.014; El Hachem, M., Zambruno, G., Bourdon-Lanoy, E., Ciasulli, A., Buisson, C., Hadj-Rabia, S. et al., 2014, ‘Multicentre consensus recommendations for skin care in inherited epidermolysis bullosa’, *Orphanet Journal of Rare Diseases* 9, 76–76. https://doi.org/10.1186/1750-1172-9-76; Saad, R., Duipmans, J., Yerlett, N., Plevey, K., Mccuaig, C., Woolfe, W. et al., 2024, ‘Neonatal epidermolysis bullosa: A clinical practice guideline’, *British Journal of Dermatology* 190(5), 636–656. https://doi.org/10.1093/bjd/ljae006

Note: General principles of preventing wound formation.

#### Consensus statement 8: We recommend regularly assessing wounds for complications such as infection and malignancy

- It is crucial to assess the type, size and extent of the wound (Pope et al. 2012).
- Vigilant monitoring for complications such as infection and SCC. The mnemonic NERDS- Non-healing lesions, Exudate, Red friable tissue, Debris on the wound base, and Smell indicate critical colonisation or infection if three or more features are present (Sibbald et al. 2006).
- Take microbial swabs from all wounds suspected of being infected. The choice of antibiotic is guided by microbial sensitivity.
- Signs of cutaneous SCC include: (i) a non-healing wound, lasting longer than normal EB wounds, that is 4 weeks or more; (ii) a rapidly growing wound with excessive granulation tissue; (iii) deep, punched-out ulcer with a raised or rolled edge; (iv) an area of hyperkeratosis and a shoulder of raised skin and (v) a wound with altered sensation (Mellerio et al. 2016).
- Dermoscopy should be conducted for patients with EB nevi and suspected SCC (El Hachem et al. 2014).

#### Consensus statement 9: We recommend creating an ideal environment for wound healing

- Effecting wound healing requires adequate nutrition, proper oxygenation and circulation, a functioning immune system and minimising mechanical forces that disrupts the process.
- Nutrition: key elements include albumin for anabolic repair, carbohydrates and fats for cellular energy and micronutrients such as selenium, vitamins A, C, thiamine, zinc, copper and iron contributing to this process (Fonder et al. 2008).
- Anaemia: often caused by blood loss, insufficient dietary intake or poor absorption; anaemia impairs wound healing. A haemoglobin target of > 10 g/dL is recommended (Liy-Wong et al. 2023).
- Hypoalbuminaemia: Albumin levels below 30 g/L indicate nutritional deficiency and hinder healing (Pope et al. 2012). Regular monitoring of albumin every 6–12 months is advised (Pope et al. 2012).

#### Consensus statement 10: We recommend that all blisters are lanced and not deroofed

- Blisters should be carefully lanced with a sterile needle at multiple points at the lowest area to allow gravity to drain them, keeping the roof intact, to serve as a natural sterile dressing (Denyer et al. 2017).
- Sterilise all equipment (scissors, tweezers, etc.) in boiling water before use.
- Remove crusts and loose skin at the edge of the lesion to reduce the risk of infection.

#### Consensus statement 11: Bathing recommendations for patients with epidermolysis bullosa

Bathing can be extremely painful, leading to poor adherence, critical colonisation, infections and antibiotic use. The pain significantly affects quality of life, potentially contributing to depression and poor nutrition (Petersen et al. 2015).

**What should be added to the bath water? (El Hachem et al. 2014; Petersen et al. 2015; Pope et al. 2012; Madhusudhan 2016; Shayegan et al. 2020):** In areas without clean water, boil and cool water before use. Bath additives can reduce pain, odour and bacterial load.

- Saltwater baths help reduce pain, decrease the need for analgesia and improve compliance, regardless of salt concentration (Petersen et al. 2015). Salt, combined with bleach or antimicrobial cleansers, further reduces infection and drainage (Petersen et al. 2015).
  ■ Babies and children: 2 tablespoons of salt (NaCl) per 5 litres of water.
  ■ Adults: 0.45 kg to 1 kg of salt in a bathtub (150 L).
- Bleach (sodium hypochlorite) is bactericidal (Shayegan et al. 2020) but should not be used for children under 1 year of age (DEBRA International).
  ■ ¼ cup (6% bleach) to half a tub (75 L) is equivalent to 0.005%, which is bactericidal and not cytotoxic or 5 ml – 10 ml per 5 L of water.
- Acetic acid (vinegar) is effective against gram-positive and gram-negative bacteria, including *Pseudomonas aeruginosa* (Madhusudhan 2016).
  ■ 1% acetic acid applied to a gauze, 5% white vinegar diluted to 0.25% – 1%
- The prolonged use of chlorhexidine could result in neurotoxicity (Denyer & Stevens 2010) and allergy. Add 5 ml – 10 ml in 5 L water.

After any bath additive, rinse with clean water and apply a moisturiser to unaffected skin to prevent pruritus.

**How often should the patient be bathed?:** Bathing frequency depends on the wound type and dressing used (El Hachem et al. 2014; Pope et al. 2012).

Denyer et al. suggested neonates should only bathe once wounds from birth trauma have healed (Denyer & Stevens 2010), although this may not be feasible for neonates who are human immunodeficiency virus (HIV)-exposed or those with prolonged maternal exposure.

In advanced dressings, bathing can occur every 2–3 days (15, 34) or even weekly (El Hachem et al. 2014).

**What is the best method to wash the skin?:** Showering tends to be more painful than bathing because of the pressure on fragile skin. Bathing has the added benefit of allowing bandages to soak off and enabling the use of bath additives (Arbuckle 2010).

If bathing is not possible, direct shower water onto a sponge to reduce pressure on fragile skin (DEBRA International).

Caution is essential to prevent slips and injury during bathing or showering.

#### Consensus statement 12: Wound care recommendations for epidermolysis bullosa

**Wound dressings for epidermolysis bullosa in low socioeconomic settings:** In neonates, dress one limb at a time to avoid trauma from the other limb when baby is moving or agitated (Denyer & Stevens 2010). Dressing choice depends on wound type (e.g., exudative, infected, hyper granulated); patient preference, cost affordability and available resources.

Dressings should be checked daily (El Hachem et al. 2014) and soaked off before bathing and changing.

Recommended dressings for both resource-limited and resource rich settings are listed in Table 5 (DEBRA International 2017; Denyer et al. 2017; El Hachem et al. 2014; Has et al. 2021; Pope et al. 2012).

**TABLE 5:** Dressings for epidermolysis bullosa wounds.

Dressing	Names of dressings	Mechanisms of action	Changes
**Lightly exudative**			
Non-adhesive silicone mesh	Mepitel Mepitel AG (added silver) Adaptic Cuticel	Allows non-traumatic removal Can apply topicals over this dressing (except the dressings with AG)	Every 3–4 days
Lipido-colloid	Urgotul Urgotul AG (added silver)	Non-traumatic	3–4 days
Hydrogel	Intrasite Intrasite comfortable	Hydrates wounds Autolytic debridement Hydrate the wound It has a cooling effect and reduces pain and pruritus.	Daily or when it becomes dry
**Exudative dressings**			
Foams	Mepilex Mepilex lite Mepilex border PolyMem	Exudative wounds It contains silicone, so it is non-adherent For digits and folds For protection	Depends on the level of exudate Up to 7 days
Hydrofibers	Aquacel	Heavily exudative wounds	3–4 days
Alginates	Kaltostat	Exudative wounds Release calcium ions that help to stop bleeding	-
Silver dressings	Mepitel AG Urgotul AG Acticoat	Antimicrobial effect No resistance	Up to 4–7 days
Resource-limited setting	Paraffin-impregnated gauze	-	Daily
	Gauze with copious amounts of lubricant (WSP, petroleum jelly)	-	Daily
	Clean cotton material and copious amounts of lubricant (WSP, petroleum jelly)	Ensure that copious amount of lubricant is used to prevent adhesion of the primary layer.	Daily
	Toilet paper and copious amounts of lubricant (WSP, petroleum jelly)	-	Daily
	Cling wrap	If no dressings are available, be careful of overheating	Daily

*Source*: Debra International, 2017, *Skin and wound care in epidermolysis bullosa*, viewed from https://www.debra-international.org/skin-and-wound-care-in-eb; Denyer, J. & Stevens, L., 2010, ‘Bathing in epidermolysis bullosa: Benefit over trauma?’, *Wounds UK* 6, 79–84; El Hachem, M., Zambruno, G., Bourdon-Lanoy, E., Ciasulli, A., Buisson, C., Hadj-Rabia, S. et al., 2014, ‘Multicentre consensus recommendations for skin care in inherited epidermolysis bullosa’, *Orphanet Journal of Rare Diseases* 9, 76–76. https://doi.org/10.1186/1750-1172-9-76; Has, C., El Hachem, M., Bučková, H., Fischer, P., Friedová, M., Greco, C. et al., 2021, ‘Practical management of epidermolysis bullosa: Consensus clinical position statement from the European reference network for rare skin diseases’, *Journal of the European Academy of Dermatology and Venereology* 35(12), 2349–2360. https://doi.org/10.1111/jdv.17629; Pope, E., Lara-Corrales, I., Mellerio, J., Martinez, A., Schultz, G., Burrell, R. et al., 2012, ‘A consensus approach to wound care in epidermolysis bullosa’, *Journal of the American Academy of Dermatology* 67(5), 904–917. https://doi.org/10.1016/j.jaad.2012.01.016

**How often should wound dressings be changed?:** Dressing change frequency depends on wound type, patient age, pain, infection and the dressings used (Pope et al. 2012; Shayegan et al. 2020). Refer to Table 5 for details. Neonates typically require daily dressing changes (Pope et al. 2012).

##### Topical therapy used

- Antibiotics
  ■ Silver sulfadiazine, mupirocin, bacitracin, fucidin and medicated honey
  ■ Use topical antibiotics on infected wounds on a rotational basis for short durations.
  ■ Monitor for antimicrobial resistance.
  ■ Be cautious with prolonged use of silver dressings and the risk of argyria (Has et al. 2021).
- Steroids: apply to granulated tissue, especially in JEB.
- Emollients: use emulsifying ointment, 50/50 mix of white soft paraffin in liquid paraffin on xerotic skin to reduce pruritus, pain and blistering (El Hachem et al. 2014).
- Sunscreen: recommended for patients with Kindler syndrome.

#### Consensus statement 13: Understanding pain mechanisms to guide analgesia choice

Pain is a debilitating symptom in all EB types, with severity linked to the conditions impact on quality of life (Pope et al. 2012). Pain is multifactorial, stemming from dermatological (blisters, erosions secondary cutaneous infection), non-dermatological (musculoskeletal, gastrointestinal, ocular) and procedural (dressing changes, bathing, blood draws) causes (Goldschneider et al. 2014). Anxiety, depression and poor nutritional state can intensify pain (Pope et al. 2012).

Pain in EB is both nociceptive (throbbing and gnawing) and neuropathic (burning, stabbing, stinging) (Has et al. 2021; Pope et al. 2012). Accurate pain scoring is crucial but can be challenging. For children over 7 years and adults, the visual analogue or numeric rating scale is used. For children under 7 years the Face, Legs, Activity, Cry, Consolation (FLACC) scale is appropriate (Has et al. 2021).

Building rapport with patients and families is key to effective pain management. This involves assessing pain type (nociceptive or neuropathic), severity and anxiety levels (Has et al. 2021). Pain management must be individualised, considering medication side effects such as opioid induced hypogonadism, osteopenia, constipation and pruritus, or tricyclic antidepressants and methadone prolonging the QT interval on the electrocardiogram (Goldschneider et al. 2014).

Pain management: Non-pharmacological and pharmacological

Pain management may include both non-pharmacological and pharmacological treatment (Table 6). For nociceptive pain options include acetaminophen, non-steroidal anti-inflammatories (NSAIDS) and morphine. Neuropathic pain is treated with gabapentin, pregabalin and tricyclic antidepressants (Pope et al. 2012). Non-pharmacological physical strategies such as movement, exercise, proper positioning, pacing, cooling devices and cognitive behavioural techniques (e.g., attentional diversion, relaxation, cognitive flexibility, visualisation, guided imagery) help with both nociceptive and neuropathic pain (Pope et al. 2012).

**TABLE 6:** Managing pain in patients with epidermolysis bullosa.

Types of pain	Sources of pain	Management strategies
Acute pain	Postoperative	Opioids, tramadol Non-steroidal anti-inflammatories Acetaminophen Patient-controlled anaesthesia (Be mindful that patients with pseudosyndactyly may not be able to operate the device.) Regional anaesthesia (depending on the type of procedure). Caution should be exercised when preparing the site of insertion not to induce blisters.
Chronic pain Recurrent pain (acute on chronic pain)	Skin and wound pain	Optimise nutrition and treat infections Cognitive behavioural therapy (CBT) Topical anaesthesia (xylocaine, 2% lidocaine, lidocaine-prilocaine) Acetaminophen Tramadol, opioids- long-term use, monitor for hypogonadism Non-steroidal anti-inflammatories Neuropathic pain Pregabalin, gabapentin Tricyclic antidepressants Anecdotal reports of the benefit of cannabinoids
	Bathing and dressings	Keep the room warm Prepare dressings in advance Soak off the bandages Add salt to the water (see Table 5) 2% – 4% sucrose in children < 2 years Acetaminophen Opioids Non-steroidal anti-inflammatories Anxiolytics Ketamine Cognitive behavioural therapy
	Gastrointestinal: Oral Oesophageal: Reflux Stricture Anal: Constipation Fissures	Sucralfate, chlorhexidine wash Treat reflux if present Dilatation and budesonide therapy Stool softeners, modify diet Sucralfate and petroleum jelly Cognitive behavioural therapy
	Musculoskeletal: Joint pain Bone pain or osteoporosis	Non-steroidal anti-inflammatories, Acetaminophen, tramadol, opioids Physiotherapy, cognitive behavioural therapy, Surgical correction Vitamin D, calcium, bisphosphonates, exercise. Medication as per joint pain. Cognitive behavioural therapy
	Ocular pain	Lubrication eye drops Non-steroidal anti-inflammatories
Procedural	Blood draw or biopsy	Topical anaesthetics Cognitive behavioural therapy
Psychological	Anticipatory pain Anxiety	Cognitive behavioural therapy Anxiolytics

*Source*: El Hachem, M., Zambruno, G., Bourdon-Lanoy, E., Ciasulli, A., Buisson, C., Hadj-Rabia, S. et al., 2014, ‘Multicentre consensus recommendations for skin care in inherited epidermolysis bullosa’, *Orphanet Journal of Rare Diseases* 9, 76–76. https://doi.org/10.1186/1750-1172-9-76; Goldschneider, K.R., Good, J., Harrop, E., Liossi, C., Lynch-Jordan, A., Martinez, A.E. et al., 2014, ‘Pain care for patients with epidermolysis bullosa: Best care practice guidelines’, *BMC Medicince* 12, 178. https://doi.org/10.1186/s12916-014-0178-2; Has, C., El Hachem, M., Bučková, H., Fischer, P., Friedová, M., Greco, C. et al., 2021, ‘Practical management of epidermolysis bullosa: Consensus clinical position statement from the European reference network for rare skin diseases’, *Journal of the European Academy of Dermatology and Venereology* 35(12), 2349–2360. https://doi.org/10.1111/jdv.17629; Pope, E., Lara-Corrales, I., Mellerio, J., Martinez, A., Schultz, G., Burrell, R. et al., 2012, ‘A consensus approach to wound care in epidermolysis bullosa’, *Journal of the American Academy of Dermatology* 67(5), 904–917. https://doi.org/10.1016/j.jaad.2012.01.016

NSAIDS, non-steroidal anti-inflammatories.

Psychological interventions can aid in reducing both acute and chronic pain. Distraction techniques, visualisation and relaxation are effective for acute pain, while cognitive behavioural therapy is useful for chronic pain (Goldschneider et al. 2014).

Topical anaesthetics should be used with caution especially on open wounds. Using them over large areas can cause seizures and methaemoglobinaemia (El Hachem et al. 2014). Eutectic mixture of local anaesthetics (EMLA) can trigger allergic reactions (Goldschneider et al. 2014).

#### Consensus statement 14: We recommend a biopsychological approach to managing pruritus

Pruritus significantly impacts on the quality of life in patients with EB, often correlating with disease severity (Pope et al. 2012). Contributing factors include xerosis and environmental conditions (dryness, heat and humidity), sweating, stress, multiple wounds, opioid use, dressing changes and overheating because of dressings (Papanikolaou et al. 2021).

Managing pruritus in EB is challenging but achievable with various treatment strategies. These focus on reducing inflammation, promoting wound healing, and addressing underlying causes. A holistic approach should include lifestyle changes, bathing recommendations, emollients, topical and systemic medications, optimised wound care and psychological support as outlined in Table 7 (El Hachem et al. 2014; Papanikolaou et al. 2021; Pope et al. 2012).

**TABLE 7:** Management of pruritus in patients with epidermolysis bullosa.

Categories	Recommendations and/or measures
Lifestyle modifications	Wear loose-fitting clothing. Use soft bedding. Avoid triggers such as excessive heat or sweating.
Moisturisers	Apply moisturisers generously and regularly. Prioritise creams, then oils, ointments and lotions.
Prescription medication	Topical options: corticosteroids, calcineurin inhibitors Oral medications: sedating antihistamines (cetirizine, diphenhydramine, hydroxyzine), corticosteroids, tricyclic antidepressants, gabapentin, pregabalin, cyclosporine, thalidomide Explore newer biological agents: dupilumab, omalizumab, secukinumab, ixekizumab or ustekinumab
Wound care	Dressings: silver, petrolatum, 3% bismuth tribromophenate Hydrogel dressings for painful wounds. Explore biosynthetic cellulose dressings, hydrogel sheets, foams, and modified absorbent pads. Caution with adhesive bandages if they increase itchiness.
Psychological support	Educate on itch-scratch cycle. Implement relaxation techniques. Cognitive behavioural therapy including mindfulness techniques.

*Source*: El Hachem, M., Zambruno, G., Bourdon-Lanoy, E., Ciasulli, A., Buisson, C., Hadj-Rabia, S. et al., 2014, ‘Multicentre consensus recommendations for skin care in inherited epidermolysis bullosa’, *Orphanet Journal of Rare Diseases* 9, 76–76. https://doi.org/10.1186/1750-1172-9-76; Papanikolaou, M., Onoufriadis, A., Mellerio, J.E., Nattkemper, L.A., Yosipovitch, G., Steinhoff, M. et al., 2021, ‘Prevalence, pathophysiology and management of itch in epidermolysis bullosa’, *British Journal of Dermatology* 184, 816–825. https://doi.org/10.1111/bjd.19496; Pope, E., Lara-Corrales, I., Mellerio, J., Martinez, A., Schultz, G., Burrell, R. et al., 2012, ‘A consensus approach to wound care in epidermolysis bullosa’, *Journal of the American Academy of Dermatology* 67(5), 904–917. https://doi.org/10.1016/j.jaad.2012.01.016

#### Consensus statement 15: Palliative care is an essential aspect of epidermolysis bullosa management

The philosophy of Paediatric Palliative Care (PPC) emphasises holistic care, fostering collaboration between families and multidisciplinary teams from diagnosis through all stages of the child’s healthcare journey. Its goal is to help children with EB live as well as possible for as long as possible. Furthermore, PPC involves assessing and alleviating physical, spiritual, psychological and social distress through skilled communication, shared decision-making, family support and a multidisciplinary approach. In this regard, DEBRA’s EB Best Practice Guidelines highlight Palliative Care Principles as a cornerstone of EB management (Goldschneider et al. 2014, Popenhagen et al. 2023).

Early planning is essential including pain management, psychosocial support and consultation with allied health professionals such as occupational therapists and dieticians. Whether a child’s life is long or short, the aim is to ensure the best quality of life.

Four key concepts in palliative care for epidermolysis bullosa:

- **Coordinated multidisciplinary approach:** holistic care using palliative care principles should begin at diagnosis. A palliative care plan can be filed in medical records and held by families (Online Appendix 1 – Table 9-A1).
- **Effective communication: communication** is critical and can be enhanced using tools such as - *St Jude’s Department of Paediatric Medicine’s Quick Communication Reference Guide* detailing strategies for delivering difficult news, responding to emotions and setting care goals.

The *Vital Talk App* provides a practical approach to running a family meeting and sharing news about withholding or withdrawing life sustaining treatments when it is no longer in the child’s best interest.

- **Ethical considerations and shared decision-making:** decisions about life-sustaining treatments such as ventilation, inotropic support and antibiotics should prioritise the child’s best interest, avoiding painful interventions that offer no clinical benefit (Larcher et al. 2015). Paediatric Advance Care Planning (pACP) aligns care with family preferences, serving as a clinical guide rather than a legal document.
- **Complicated grief:** Families, including siblings, are at high risk for complicated grief because of EB’s mortality rates. Connecting families to grief and bereavement support is vital. Remote or in-person counselling is recommended.

Palliative care ensures compassionate, family-centred support throughout the EB journey, addressing the multifaceted challenges posed by the condition.

Contact details to assist with care include the following:

- Paediatric Palliative Care Specialist Location
  Palliative Care for Children South Africa (PatchSA) (www.patchsa.org)
  Association of Palliative Care Practitioners of South Africa (www.palprac.org)
- National grief support organisation: The Compassionate Friends
  Telephone: (011) 440-6322 or 084 332-1876
http://www.compassionatefriends.co.za/

#### Consensus statement 16: Genetic counselling is essential to holistic care for patients and families

Genetic counselling and testing are critical for preventing disease recurrence and ensuring comprehensive care patients and families.

**Explaining the disease mechanism and gene(s) involved:** Educating families about the disease mechanism and the genetic inheritance is vital. Using graphical representations can enhance understanding, helping families grasp the diagnosis, adjust emotionally and accept the treatment plan (Chong et al. 2021).

**Setting realistic expectations:** While EB prognosis varies with subtypes, some severe forms are associated with high mortality (Chong et al. 2021); hence, compassionate communication about the child’s specific EB type is necessary, providing families with realistic expectations and emotional support if the prognosis is poor.

**Emotional support and palliative care:** Genetic counselling must include emotional and logistical support. Referral’s to social work and psychological services may be required for ongoing assistance. Palliative care organisations such as Palliative Care for Children South Africa (PatchSA) and Umduduzi Children’s Hospice can assist with support and planning for the family.

**Recurrence risk and family implications:** Genetic testing is vital. For autosomal dominant EB: if the parents are unaffected, the recurrence risk is typically estimated at ±1% due to possible gonadal mosaicism. Affected individuals surviving into adulthood have a 50% chance of passing their condition to their offspring. Autosomal recessive EB: parents are obligate carriers with a 25% recurrence risk per shared pregnancy. Patients siblings may also be carriers with similar risk of transmitting the condition depending on their partners’ genetic status.

## Recommendations

- Establish centres of excellence for rapid referral, diagnostics, prevention and management of complications and multidisciplinary care of patients (Chateau et al. 2024b).
- There should be continuous education provided to HCPs to ensure rapid diagnosis and referral to specialist centres (Chateau et al. 2024b).
- Encourage collaboration with other HCPs caring for patients with EB.
- The principal investigator has observed that most Zulu-African patients with EB in KwaZulu-Natal, SA have JEB with mutations in *LAMB3*. This allows for development of affordable gene panels that will allow rapid diagnosis of patients in our setting.
- There should be collaboration with pharmaceutical companies and medical aids to ensure affordable dressings and treatment for patients.
- Diagnostics should be readily available in government hospital settings to allow for rapid diagnostics, management and prognostication of patients.

## Conclusion

Currently, no comprehensive care guidelines or consensus statements exist for managing patients with EB in Africa. This document provides a multidisciplinary framework for the holistic care of EB patients and their families considering resource constraints and cultural diversity.
